# Participation of patients during arthroscopic partial meniscectomy is conducive to postoperative rehabilitation and satisfaction: a single-center retrospective study

**DOI:** 10.1186/s12891-022-05778-9

**Published:** 2022-09-02

**Authors:** Pengfei Ruan, RuiQing Ji, Jing Shen, Xiang Wang, Weifeng Ji

**Affiliations:** 1 Zhejiang Chinese Medical University, Hangzhou, China; 2 Department of Anaesthesia, The Second Clinical Medical College of WenZhou Medical University, WenZhou, China; 3 The First Affiliated Hospital of Zhejiang Chinese Medical University, Hangzhou, China; 4 Department of Orthopaedics, Tongde Hospital of Zhejiang Province, Hangzhou, China

**Keywords:** Arthroscopic partial meniscectomy, Intraoperative participation, Rehabilitation

## Abstract

**Purpose:**

To evaluate the effect of patient participation in arthroscopic partial meniscectomy (APM) on rehabilitation and patient satisfaction.

**Methods:**

A total of 86 patients of traumatic longitudinal vertical meniscus tears, between January 2017 and December 2020 at the First Affiliated Hospital of Zhejiang Chinese Medical University, met the inclusion and exclusion criteria. The patients in the intraoperative participation group (*n* = 33) were awake and could watch the screen during APM and communicate with the surgeon in the surgery; patients who underwent APM in the traditional mode were classified as the traditional group(*n* = 53). The differences in exercise adherence, the Knee Injury and Osteoarthritis Outcome Score (KOOS) and satisfaction at follow-up were compared. In the intraoperative participation group, the mean age of the patients was 26.97 ± 5.63 years and the follow-up time was 25.12 ± 6.23 months. In the traditional group, the mean age of the patients was 29.21 ± 5.29 years and the follow-up time was 25.08 ± 6.70 months.

**Results:**

The intraoperative participation group reported a better result in exercise adherence (78.79% VS 50.94%, *p* = 0.012). As secondary outcomes, Patients in the intraoperative participation group demonstrated better scores on the KOOS domains of pain (79.80 ± 6.38 VS 76.26 ± 5.33, *p* = 0.007), Symptoms (59.41 ± 5.27 VS 56.74 ± 5.97, *p* = 0.038), and QOL (65.91 ± 10.72 VS 60.26 ± 9.34, *p* = 0.012), as compared to these in the traditional group. There were no significant differences in the KOOS domains of Sport (72.88 ± 8.20 VS 72.64 ± 7.70, *P* = 0.892), and ADL (89.47 ± 3.50 VS 87.87 ± 4.50 *p* = 0.085). what’s more, in the intraoperative participation group, the results of satisfaction (96.97% VS 81.13%, *p* = 0.025) were also significantly better.

**Conclusion:**

The mode of participation of patients during APM can improve patients’ exercise adherence, reduce pain, improve symptoms and improve patients’ satisfaction as well as the quality of life. More work is needed to develop this mode further.

## Introduction

Since Ikeuchi performed the first arthroscopic repair in 1969, arthroscopy has shown its vigorous vitality with the development of various arthroscopic techniques [1]. Take arthroscopic partial meniscectomy (APM) as an example, the rate of APM more than doubled in the UK between 1997 and 2017 [2].

Postoperative exercise is a subset of physical activity that is planned, structured, and repetitive, to improve or maintain physical health [3], and it is one of the favorable factors for the improvement of patients who have undergone APM [4]. Patients with exercise adherence have better outcomes than non-adherence patients [5, 6], and poor adherence has implications on treatment cost and effectiveness [7]. Although the benefits of postoperative exercise are known, the exercise adherence of patients is still one of the concerns of postoperative rehabilitation [8, 9], and the rate of patients with non-adherence was 50%-70% [10].

Many surgeons investigated risk factors that influence patient adherence to exercise [9–13], The confidence that exercise could affect pain relief and functional improvement plays an important role in exercise adherence [10, 14]. We propose a new operation mode in which patients could watch the screen and commune with the surgeon during arthroscopic surgery. And we will record the procedure and give it to the patient after the operation. In this way, patients can participate in the surgery, and they can even make their own demands. If patients require it, we would do the arthroscopic surgery in this mode. We considered that participation in the surgical procedure could enhance the confidence of patients, encourage postoperative exercise, improve the exercise adherence of patients and promote their postoperative recovery. In this retrospective study, we investigated exercise adherence and postoperative recovery after APM in our hospital to evaluate the improvement of patients’ participation during APM. We hypothesized that participation of Patients During APM would lead to better exercise adherence, as well as higher the Knee Injury and Osteoarthritis Outcome Score (KOOS) and satisfaction.

## Methods

### Patient selected

The study design was a retrospective study, which was approved by the ethics committee of our hospital. To eliminate interference, the inclusion criteria were (1) a history of an acute knee injury and (2) verified a longitudinal vertical tear on both MRI and knee arthroscopy. And patients older than 35 years [15], with ligament injuries or a history of other lower extremity surgery/trauma were excluded. A total of 86 patients treated in our hospital between January 2017 and December 2020 were eventually followed up in the study. All patients were invited to the clinic for follow-up. Even if a proportion of patients couldn’t come to the clinic, we followed up with those patients by email or direct telephone.

As a routine procedure, all patients underwent APM under combined spinal-epidural anesthesia (CSEA). By browsing the medical records, patients who underwent APM in the traditional mode were classified as the traditional groups. Patients who participated in APM were classified as the intraoperative participation group.

In the traditional group, patients would be sedated intravenously. Therefore, they couldn’t communicate with the surgeon during APM. Instead, the patients in the intraoperative participation group were awake and could watch the screen during APM. In this way, communication with the surgeon in the surgery was encouraged. Of course, patients in this group could put forward some requirements during surgery, such as “Retain more meniscus and synovium”. However, the patients’ intervention is relatively small. We would not meet their requirements if unreasonable, but instead, tell them why we did so.

All patients would receive health education and rehabilitation guidance from the same physiotherapist on the first day after the operation. The health education emphasized the importance of rehabilitation exercise for reducing pain and improving function. The rehabilitation program is mainly for knee flexion and extension in a non weight-bearing position, and the appropriate exercise intensity and frequency are selected according to the tolerance of the muscles around the joint. Therefore, we could evaluate the exercise adherence of patients through the completion of their rehabilitation program.

### Clinical assessment

The medical records of the 86 patients meeting the inclusion and exclusion criteria were reviewed in pre-operation and discharge. And patients were followed up by being invited to the clinic, or by direct e-mail or telephone. Medical records of traumatic meniscus tears were reviewed by a single experienced surgeon. The patient’s symptoms were confirmed to be due to traumatic meniscus tears.

### Evaluated parameters

The data recorded in the medical records include baseline data and drug use. In pre-operation, we recorded the baseline data, such as age, sex, body mass index, and education level. The outcomes were collected by clinic, email or direct telephone through the questionnaires. As primary outcomes, exercise adherence was recorded as follows. We would make an appropriate rehabilitation program for the patients, and those who adhered to < 50% of their program elements, including exercise intensity and frequency, as non-adherent [9].

We also compared satisfaction and the Knee Injury and Osteoarthritis Outcome Score (KOOS) at follow-up as secondary outcomes. The KOOS is a questionnaire to assess outcomes in patients undergoing meniscus surgery over short- and long-term follow-up (one week to decades). The score ranges from 0 (extreme symptoms) to 100 (no symptoms), with five different sub-scores, including symptoms, pain, sport, activities of daily living (ADL), and quality of life (QOL) [16]. Therefore, the higher KOOS scores mean better postoperative rehabilitation in this domain. For satisfaction, patients responded to the following questions: “Are you satisfied with your knee after the surgery?” on a 5-point Likert scale. We would get a reply, including “Very satisfied”, “Satisfied”, “Neither satisfied nor dissatisfied”, “Dissatisfied” and “Very dissatisfied”. We classified “Very satisfied” and “Satisfied” as satisfied, and “Neither satisfied nor dissatisfied”, “Dissatisfied” and “Very dissatisfied” as dissatisfied [17].

### Statistical analysis

Analysis was performed using SPSS Statistics software version 20.0(IBM, Armonk, NY). Continuous data were described using means, standard deviations, and 95% confidence intervals (CIs), whereas categorical data were described as frequencies and percentages. A χ2 test was used to compare the categorical variables. A two-sample *t*-test was used to compare the continuous variables between the groups. The significance level was set at 0.05.

## Results

**Fig. 1:**
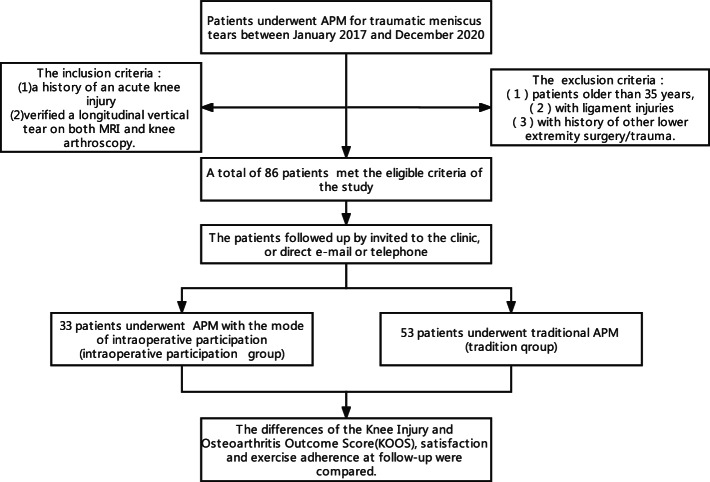
Study flow chart

Finally, among the 86 patients who met the eligible criteria of the study. 33 patients chose the mode of intraoperative participation, while others received traditional surgical treatment (Fig. 1). The baseline characteristics of the included patients are shown in Table 1. There was no significant difference in age, sex, body mass index, education level and follow-up time (Table 1).

**Table 1:** Baseline characteristics of total 86 patients

Data	Intraoperative participation group (*n* = 33)	Traditional group (*n* = 53)	*P* value
Age(year)	26.97 ± 5.63(24.97–28.97)	29.21 ± 5.29(27.75–30.67)	0.066
Sex(F/M)	13/20	19/34	0.820
BMI(kg/m^2^)	23.95 ± 2.79(22.96–24.94)	23.38 ± 3.36(22.46–24.31)	0.417
Education level			
High school graduate or Less	14	28	0.298
In college	5	3	
College graduate	14	22	
Follow-up time	25.12 ± 6.23(22.91–27.33)	25.08 ± 6.70(23.29–26.92)	0.975

*BMI* Body mass index

In the analysis, patients in the intraoperative participation group showed better results in exercise adherence and satisfaction (Table 2), and the intraoperative participation group had a better score on the KOOS domains of pain, symptoms and quality of life (Table 3). We did not find any serious adverse events.

**Table 2:** Secondary outcomes at follow-up

Outcome	Intraoperative participation group (*n* = 33)	Traditional group (*n* = 53)	*P* value
The rate of adherencers	26/33(78.79%)	27/53(50.94%)	0.012*
The rate of satisfaction	32/33(96.97%)	42/53(81.13%)	0.025*
Serious adverse events	0	0	

^*^Statistically significant

**Table 3:** The Knee Injury and osteoarthritis outcome scores at follow-up

Outcome	Intraoperative participation group (*n* = 33)	Traditional group (*n* = 53)	*P* value
KOOS Pain	79.80 ± 6.38(77.53–82.06)	76.26 ± 5.33(74.79–77.73)	0.007*
KOOS Symptoms	59.41 ± 5.27(57.55–61.28)	56.74 ± 5.97(55.09–58.38)	0.038*
KOOS ADL	89.47 ± 3.50(88.23–90.72)	87.87 ± 4.50(86.63–89.11)	0.085
KOOS Sport	72.88 ± 8.20(69.97–75.79)	72.64 ± 7.70(70.52–74.76)	0.892
KOOS QOL	65.91 ± 10.72(62.11–69.71)	60.26 ± 9.34(57.58–62.83)	0.012*

KOOS the Knee Injury and Osteoarthritis Outcome Score

*ADL* Activities of daily living, *QOL* Quality of life

^*^Statistically significant

## Discussion

We found the patients in the intraoperative participation group had better exercise adherence. What’s more, the patients in the intraoperative participation group have a significantly larger improvement in scores on the KOOS domains of pain, symptoms and quality of life—indicating better patient-reported outcomes—than those in the traditional group. It shows that participation of patients during APM is beneficial for urging patients to exercise and postoperative recovery.

Rehabilitation after arthroscopy is a noteworthy aspect. Exercise adherence is important for postoperative rehabilitation. The meta-analysis by Pan et al. [18] showed that medical exercise therapy combined with APM is effective in reducing pain and improving range of motion in the early postoperative period. Strengthening exercises have been shown to be effective treatments for reducing pain and improving function in patients with meniscus tears or knee OA [19]. However, some patients become non-adherent to their exercise program. The reasons for non-adherence are complex, involving the willingness and ability to accommodate exercises in daily life, the perceived severity of symptoms, the desire to get back to usual activities and increased pain levels during exercise [8, 9, 11]. We consider that participation of patients during APM can help patients understand the effectiveness of the APM and have better exercise adherence, which is proved by the results.

It is likely that psychological factors of patients significantly influence their rehabilitation after the surgery to the extremity orthopaedic extremities. Some studies on lower extremity surgery have found an association between high preoperative expectations and better outcomes [20–22]. For example, Henry et al. [22] reported that a higher expectation may be associated with better activity and less pain after extremity orthopaedic surgery. We consider that high preoperative expectations may increase exercise adherence by patients, to help with postoperative recovery. In Pinsornsak et al.’s randomized controlled trial [23], the use of immediate post-operative knee range of motion photographs resulted in enhancing early knee flexion and function at six weeks. It’s also proved that higher expectation mean better postoperative recovery. Participation of patients during APM can also improve expectations, and be an incentive for actively carrying out rehabilitation exercises. In this way, patients could show better outcomes, such as less pain and symptoms.

In this study, patients in the intraoperative participation group were more satisfied. It is well recognized in the service industries that satisfaction is the most important criterion of success. In the meantime, it has also been used as a healthcare performance indicator for surgery [24]. Mira et al. [25] found that not only successful surgical procedures but also the experience of surgery all substantially influenced the patient’s overall satisfaction response. According to the cross-sectional surgery patients’ survey in 24 public hospitals in Spain and a total of 15,539 inpatients and 7,899 outpatients conducted by Hamilton et al. [24], overall patient’s satisfaction following joint arthroplasty is significantly affected by fulfillment of presurgical expectations, symptomatic pain relief achieved following surgery and the hospital experience. Therefore, patients’ experience in the hospital is an important factor of satisfaction. Through intraoperative participation, patients can better understand the operation process and more trust in surgeons, so as to improve their satisfaction.

### Advantages and disadvantages

Obviously, the mode of intraoperative participation can improve patients’ exercise adherence and postoperative recovery. What’s more, this mode is easy to implement without any new tools.

Distracting the surgeon may be one of the disadvantages of this mode. Due to this, the operation time may be prolonged, resulting in some complications. However, we did not find any serious adverse events in this study.

### Limitations

Several limitations of our study must be considered. First, our study is a single-center design with a relatively low number of patients. Secondly, Meniscus repair is more recommended for acute tears. However, due to hospitalization expenses and higher reoperation in the short-term, most patients chose APM in our hospital. Thirdly, our study included patients with a traumatic longitudinal vertical tear only, which means this mode may not have a good effect on other tear types. Moreover, we did not formally assess the psychological impact of intraoperative participation between patients and surgeons.

## Conclusion

The mode of participation of patients during APM can improve patients’ exercise adherence, reduce pain, improve symptoms and improve patients’ satisfaction as well as quality of life. More work is needed to develop this mode further.

## Data Availability

The datasets used and analysed during the current study are available from the corresponding author on reasonable request.
